# Supplementary material for: Longitudinal impact of screening colonoscopy on greenhouse gas emissions
Source: PLoS One. 2024 Jul 19;19(7):e0307133. doi: 10.1371/journal.pone.0307133 (PMC11259268; doi:10.1371/journal.pone.0307133)
Supplement: Attachment — Submitted filename: PLOS ONE, Mayo-2024.pdf [file pone.0307133.s001.pdf]

Dear authors,

I found your article to be very well-written, original, and highly relevant given the current times. It is very well-structured and thought out. I just wanted to mention a few points:

- On page 5, you mention that group 2 of patients included in the NordICC trial was not offered screening... This seems unethical to me; those individuals were deprived of the opportunity for detection.

- On page 7, you mention that approval was not required... Why is that?

- On page 14, where the study limitations are listed, we should also consider transfers due to treatment complications (hospitalizations, extra trips to be evaluated by their doctors for the presence of uncommon symptoms, etc.).
